# Relationship between body composition and pulmonary function in the general population—a cross-sectional study in Ningxia

**DOI:** 10.1038/s41598-023-44486-9

**Published:** 2023-10-19

**Authors:** Yang-yang Pi, Wen-xuan Hu, Zi-ming Jiao, Peng-yi Hou, Yu-hong Zhang, Yi Zhao, Xiao-xia Li, Jing Yu, Fang Chen, Jin-yun Jing, Fa-xuan Wang

**Affiliations:** 1 NHC Key Laboratory of Metabolic Cardiovascular Diseases Research, Ningxia Medical University, Yinchuan, 750004 People’s Republic of China; 2 School of Public Health, Ningxia Medical University, Yinchuan, 750004 People’s Republic of China; 3 Ningxia Hui Autonomous Region Maternal and Child Health Care Hospital, Yinchuan, 750004 People’s Republic of China

**Keywords:** Diseases, Health care, Medical research, Risk factors

## Abstract

Studies considering the relationship between non-obesity-related body composition and lung function are few; therefore, this study aimed to explore these correlations and effects. This cross-sectional study conducted in rural Qingtongxia City and Pingluo County, Ningxia, China, included 776 participants aged 30–75 years. Body composition and lung function were measured using direct segmental multifrequency bioelectrical impedance analysis and a digital spirometer, respectively. Their correlation was assessed using partial correlation analysis, controlling for age and smoking status, and the body composition effect on lung function was analyzed using binomial logistic regression analysis. The body components total body water content, protein content, mineral content, muscle mass, fat-free mass (FFM), skeletal muscle mass, basal metabolic volume, and chest circumference (CC) positively correlated with pulmonary function (forced vital capacity and forced expiratory volume in one second) in both sexes. Neck circumference and hip circumference positively correlated with pulmonary function in women. Additionally, lung function declines more slowly in women (odds ratio [OR] = 0.66, 95% confidence interval [CI] = 0.44–0.98, *p* =  0.04); CC (OR = 0.92, 95% CI = 0.86–0.98, *p* = 0.01) increased as a protective factor for decreased lung function. Increased waist circumference (OR = 1.04, 95% CI = 1.00–1.09, *p* = 0.04) was a risk factor for reduced lung function. FFM contains body composition indicators positively correlating with lung function, excluding fat-related body composition. Abdominal obesity increases the risk of decreased lung function.

Pulmonary function is a long-term predictor of all-cause and cardiovascular mortality in the general population^1–3^ and can be influenced by various genetic or environmental factors, including malnutrition, recurrent infections, and exposure to toxic substances^4^. The human body mainly consists of water, fat, protein, and minerals, and body fat is an important predictor of morbidity and mortality from various diseases^5^.

Many studies have reported that body composition affects lung function in specific populations^6–8^, and they have often used a two-compartment model^9^, dividing body composition into fat mass (FM) and fat-free mass (FFM). Several studies have focused on body size and obesity-related effects on lung function; for example, studies using body mass index (BMI) as an indicator of body size have reported that a relatively high BMI is associated with pulmonary dysfunction^10^. Waist circumference (WC) and waist-to-hip ratio (WHR), as measures of central obesity, have been reported to be negatively correlated with lung function^11^. Several studies have identified a negative correlation between lung function and FM but a positive correlation with FFM^12,13^. Furthermore, upper body fat distribution may negatively correlate with lung volume and capacity^14^.

However, few studies have considered the relationship between the indicators included in the FFM and pulmonary function in participants, in addition to BMI and fat. With the development of anthropometry, obtaining the composition of the human body in several ways has become possible^15^, although because of radiation exposure, technical complexity, and cost, some methods are unsuitable for large-scale and general population surveys^16^. Bioelectrical impedance analysis (BIA) predicts body composition based on the conductive properties of the body^17^. It is easy to perform, safe, painless, economical, highly accurate, and has promising applications^18^. Direct segmental multi-frequency bioelectrical impedance analysis (DSM-BIA**)** improves the accuracy of moisture and fat^19^.

Based on a cross-sectional survey, this study aimed to measure body composition and lung function in participants, comprehensively analyze the correlation between body composition indicators and lung function, and explore the effect of body composition on pulmonary function in a larger population, which could provide a basis for the prevention and improvement of lung disease.

## Results

Overall, 776 participants (Qingtongxia (*n* = 565) and Pingluo County (*n* = 212)) were included in this study; of the participants, 314 were men and 462 were women. The overall mean age was 54.6 ± 8 years. Moreover, 146 (18.81%) were current smokers, and 140 (44.59%) were men. A total of 456 women (98.7%) had never smoked. The results revealed that age, height, weight, total body water content (TBW), protein content (PC), mineral content (MC), MM, FFM, skeletal muscle mass (SMM), basal metabolic volume (BMR), chest circumference (CC), WC, neck circumference, hip circumference, forced vital capacity (FVC), and forced expiratory volume in one second (FEV1) were all significantly higher in men than in women (*p* < 0.001), whereas FM, body fat percentage (BFP), and visceral fat level (VFL) were significantly higher in women than in men (*p* < 0.001). The differences in BMI, WHR, and FEV1/FVC were not statistically significant between sexes (*p* > 0.05) (Table 1).

**Table 1:** Comparison of body composition and pulmonary function indicators between men and women. (1) TBW—total body water content(L), PC—protein content(kg), MC—mineral content(kg), FM—fat mass(kg), MM—muscle content(kg), FFM—fat-free mass(kg), SMM—skeletal muscle mass(kg), BMI—body mass index(kg/m^2^), BFP—body fat percentage(%), WHR—waist to hip ratio, VFL—visceral fat level, BMR—basal metabolic volume(kcal), CC—chest circumference(cm), WC—waist circumference(cm), NC—neck circumference(cm), HC—hip circumference(cm). FVC-exertional lung volume, FEV1-exertional expiratory volume in one second, FEV1/FVC-ratio of first second exertional volume to exertional lung volume. FVC- forced vital capacity(L), FEV1- forced expiratory volume in one second(L), FEV1/FVC- the ratio of forced expiratory volume in one second to forced vital capacity. (2) The normally distributed data: mean (SD); Non-normally distributed data : median (Q1,Q3); (4) Smoking status : n (%). (5)*P ≤ 0.05, **P ≤ 0.01, ***P ≤ 0.001.

	Overall (*N* = 776)	Men (*N* = 314)	Women (*N* = 462)	*p*
Age(years)	54.6 (8.0)	56.3 (8.4)	53.5 (7.5)	0.001***
Smoking status, n(%) never	612 (78.87)	156 (49.68)	456 (98.70)	0.001***
past	18 (2.32)	18 (5.73)	0 (0.00)	
current	146 (18.81)	140 (44.59)	6 (1.30)	
Height(cm)	160.3 (7.9)	166.7 (5.6)	156.0 (6.2)	0.001***
Weight(kg)	65.2 (10.3)	70.3 (10.4)	61.8 (8.7)	0.001***
TBW(L)	31.7 [28.4, 36.8]	37.5 [35.1, 40.6]	29.1 [27.1, 31.4]	0.001***
PC(kg)	8.4 [7.5, 9.8]	10.0 [9.3, 10.8]	7.7 [7.2, 8.3]	0.001***
MC(kg)	2.9 [2.6, 3.2]	3.3 [3.1, 3.6]	2.7 [2.4, 2.8]	0.001***
FM(kg)	20.9 (6.6)	19.1 (6.8)	22.2 (6.1)	0.001***
MM(kg)	40.6 [36.4, 47.2]	48.1 [45.0, 52.1]	37.3 [34.7, 40.2]	0.001***
FFM(kg)	42.9 [38.5, 49.8]	50.8 [47.5, 55.0]	39.5 [36.7, 42.5]	0.001***
SMM(kg)	23.3 [20.7, 27.5]	28.2 [26.3, 30.7]	21.3 [19.6, 23.0]	0.001***
BMI(kg/m^2^)	25.3 (3.4)	25.3 (3.3)	25.4 (3.4)	0.599
BFP(%)	32.6 [26.7, 37.2]	27.0 [21.5, 31.2]	35.7 [32.2, 39.7]	0.001***
WHR	0.9 (0.1)	0.9 (0.1)	0.9 (0.1)	0.097
VFL	9.9 (3.9)	8.4 (3.6)	10.9 (3.7)	0.001***
BMR(kcal)	1297.5[1201.2,1446.2]	1468.5[1396.8,1557.0]	1223.5[1163.0,1288.0]	0.001***
CC(cm)	94.6 (6.7)	98.1 (6.3)	92.3 (5.9)	0.001***
WC(cm)	88.2 (9.6)	89.9 (10.6)	87.0 (8.6)	0.001***
NC(cm)	37.4 (2.9)	38.9 (2.4)	36.4 (2.7)	0.001***
HC(cm)	95.1 (5.1)	96.5 (5.1)	94.2 (4.9)	0.001***
FVC(L)	2.4 (0.6)	2.8 (0.6)	2.1 (0.5)	0.001***
FEV1(L)	2.0 (0.5)	2.3 (0.5)	1.7 (0.4)	0.001***
FEV1/FVC	0.8 [0.8, 0.9]	0.9 [0.8, 0.9]	0.8 [0.8, 0.9]	0.205

To control for the confounding effect of sex, the participants were divided into two groups based on sex (male and female), and the relationship between body composition indices and lung function was compared separately. Overall, 191 men demonstrated reduced lung function (total prevalence, 60.83%), 123 had normal lung function, 275 women had decreased pulmonary function (total prevalence, 59.52%), and 187 had normal pulmonary function. The number of participants in the FVC decline group was 404 (prevalence, 52.06%), of whom 170 were men (prevalence, 54.14%) and 234 were women (prevalence, 50.65%), while the number of participants in the FEV1 decline group was 386 (prevalence, 49.74%), of whom 142 were men (prevalence 45.22%) and 244 were women (prevalence 52.81%). The FEV1/FVC decline group comprised seven participants (prevalence, 0.9%), of whom one was male (prevalence, 0.32%) and six were female (prevalence, 1.30%). The MC in men was significantly different between the normal and decreased FVC groups, whereas the remaining body composition indicators were not significantly different between the normal and decreased pulmonary function indicator groups. Age was significantly different between the normal and declining FVC and FEV1 groups in men (Table 2). BMI and HC were significantly higher in the female FVC normal group than in the FVC decreased group. BMI, BFP, and HC were significantly higher in the FEV1 normal group than in the decreased group, whereas the remaining body composition indicators were not significantly different between the normal and decreased pulmonary function indicator groups. There was a difference in age between the normal and declining FVC groups in women (Table 3).

**Table 2:** Comparison of body composition indicators between the normal and decreased lung function groups in men**.** (1)Visceral fat levels(VFL) were classified into two types expressed as vfl: 1: normal (VFL < 10); 2: visceral fat distribution (VFL ≥ 10). vfl was expressed as a proportion (%). (2)Non-normal data log-transformed for comparison, mean (sd), median (Q1,Q3)**.** (3)*P ≤ 0.05, **P ≤ 0.01, ***P ≤ 0.001.

*n*	FVC		*p*	FEV1		*p*	FEV1/FVC		*p*
	Norml (144)	Descent (170)		Norml (172)	Descent (142)		Normal (313)	Descent (1)	
TBW	1.6 (0.0)	1.6 (0.0)	0.071	1.6 (0.0)	1.6 (0.1)	0.931	1.6 (0.0)	1.6 (NA)	NA
PC	1.0 (0.0)	1.0 (0.0)	0.078	1.0 (0.0)	1.0 (0.1)	0.905	1.0 (0.0)	1.0 (NA)	NA
MC	0.5 (0.1)	0.5 (0.1)	0.035*	0.5 (0.1)	0.5 (0.1)	0.908	0.5 (0.1)	0.5 (NA)	NA
FM	18.9 (6.7)	19.2 (6.9)	0.724	19.1 (6.6)	19.1 (7.0)	0.978	19.1 (6.8)	23.9 (NA)	NA
MM	1.7 (0.0)	1.7 (0.0)	0.075	1.7 (0.0)	1.7 (0.1)	0.914	1.7 (0.0)	1.7 (NA)	NA
FFM	1.7 (0.0)	1.7 (0.0)	0.069	1.7 (0.0)	1.7 (0.1)	0.931	1.7 (0.0)	1.7 (NA)	NA
SMM	1.5 (0.1)	1.4 (0.1)	0.098	1.4 (0.1)	1.5 (0.1)	0.835	1.5 (0.1)	1.4 (NA)	NA
BMI	25.2 (3.2)	25.3 (3.4)	0.895	25.4 (3.2)	25.2 (3.4)	0.635	25.3 (3.3)	25.0 (NA)	NA
BFP	26.9 [21.0,31.1]	27.4 [22.4, 31.7]	0.445	26.9 [21.7,31.1]	27.4 [21.3, 31.4]	0.912	26.9 [21.5, 31.2]	32.6 [32.6, 32.6]	0.26
WHR	0.9 (0.1)	0.9 (0.1)	0.83	0.9 (0.1)	0.9 (0.1)	0.471	0.9 (0.1)	1.0 (NA)	NA
vfl 1	97 (67.4)	112 (65.9)	0.875	118 (68.6)	91 (64.1)	0.469	209 (66.8)	0(0.0)	0.725
2	47 (32.6)	58 (34.1)		54(31.4)	51 (35.9)		104 (33.2)	1 (100.0)	
BMR	3.2 (0.0)	3.2 (0.0)	0.07	3.2 (0.0)	3.2 (0.0)	0.914	3.2 (0.0)	3.2 (NA)	NA
CC	98.3 (5.9)	97.9 (6.6)	0.581	98.1 (6.1)	98.1 (6.5)	0.936	98.1 (6.3)	97.6 (NA)	NA
WC	89.9 (10.6)	89.9 (10.7)	0.977	89.7 (10.5)	90.2 (10.8)	0.687	89.9 (10.7)	95.6 (NA)	NA
NC	38.9 (2.2)	38.9 (2.6)	0.92	38.8 (2.3)	39.0 (2.6)	0.416	38.9 (2.4)	39.1 (NA)	NA
HC	96.6 (5.0)	96.4 (5.3)	0.71	96.5 (5.0)	96.4 (5.3)	0.837	96.5 (5.1)	96.4 (NA)	NA
Age	57.4(8.3)	55.5 (8.4)	0.049*	57.7 ( 8.4)	54.8 (8.1)	0.002**	56.3 (8.4)	66(NA)	0.248

**Table 3:** Comparison of body composition indicators between the normal and decreased lung function groups in women**.** (1)Visceral fat levels(VFL) were classified into two types expressed as vfl: 1: normal (VFL < 10); 2: visceral fat distribution (VFL ≥ 10). vfl was expressed as a proportion (%). (2)Non-normal data log-transformed for comparison, mean (SD), median (Q1,Q3). (3)*P ≤ 0.05, **P ≤ 0.01, ***P ≤ 0.001.

*n*	FVC		*p*	FEV1		*p*	FEV1/FVC		*p*
	Normal (228)	Descent (234)		Normal (218)	Descent (244)		Normal (456)	Descent (6)	
TBW	1.5 (0.0)	1.5 (0.0)	0.299	1.5 (0.0)	1.5 (0.1)	0.752	1.5 (0.0)	1.5 (0.0)	0.5
PC	0.9 (0.0)	0.9 (0.1)	0.316	0.9 (0.0)	0.9 (0.1)	0.701	0.9 (0.0)	0.9 (0.0)	0.546
MC	0.4 (0.1)	0.4 (0.1)	0.472	0.4 (0.1)	0.4 (0.1)	0.481	0.4 (0.1)	0.4 (0.0)	0.657
FM	22.7(5.8)	21.7 (6.4)	0.093	22.8(5.4)	21.7 (6.7)	0.066	22.2(6.1)	21.9 (7.1)	0.9
MM	1.6 (0.0)	1.6 (0.0)	0.307	1.6 (0.0)	1.6 (0.1)	0.744	1.6 (0.0)	1.6 (0.0)	0.504
FFM	1.6 (0.0)	1.6 (0.0)	0.315	1.6 (0.0)	1.6 (0.1)	0.716	1.6 (0.0)	1.6 (0.0)	0.52
SMM	1.3 (0.1)	1.3 (0.1)	0.332	1.3 (0.1)	1.3 (0.1)	0.76	1.3 (0.1)	1.3 (0.0)	0.59
BMI	25.7(3.3)	25.1 (3.5)	0.043*	25.9(3.0)	25.0 (3.7)	0.005**	25.4(3.4)	24.9 (3.8)	0.735
BFP	36.0 [32.6,40.0]	35.2 [31.5,39.1]	0.198	36.3 [32.9,40.1]	35.1 [31.5,38.8]	0.021*	35.7 [32.3,39.7]	33.5 [30.5,37.2]	0.522
WHR	0.9 (0.1)	0.9 (0.1)	0.909	0.9 (0.0)	0.9 (0.1)	0.93	0.9 (0.1)	0.9 (0.1)	0.376
vfl 1	82 (36.0)	91 (38.9)	0.58	73 (33.5)	100(41.0)	0.117	170(37.3)	3 (50.0)	0.83
2	146(64.0)	143 (61.1)		145 (66.5)	144 (59.0)		286 (62.7)	3 (50.0)	
BMR	3.1 (0.0)	3.1 (0.0)	0.319	3.1 (0.0)	3.1 (0.0)	0.693	3.1 (0.0)	3.1 (0.0)	0.537
CC	92.7 (5.8)	91.8 (5.9)	0.096	92.8 (5.3)	91.8 (6.3)	0.06	92.3 (5.9)	92.3 (6.4)	0.989
WC	87.5 (8.4)	86.6 (8.7)	0.24	87.5 (7.7)	86.7 (9.3)	0.295	87.0 (8.5)	88.0(11.3)	0.789
NC	36.6 (2.7)	36.2 (2.6)	0.065	36.6 (2.5)	36.2 (2.8)	0.071	36.4 (2.7)	37.1 (3.8)	0.522
HC	94.7 (4.8)	93.8 (5.1)	0.039*	94.8 (4.4)	93.7 (5.3)	0.024*	94.2 (4.9)	93.4 (4.9)	0.671
Age	54.5(7.7)	52.5(7.3)	0.005**	54.2( 7.6)	52.9( 7.5)	0.06	53.4(7.5)	57.3(10.7)	0.208

Age and smoking status were used as control variables to analyze the correlation between body composition indices and FVC, FEV1, and FEV1/FVC using Pearson’s or Spearman’s partial correlation analysis. The results revealed that TBW, PC, MC, MM, FFM, SMM, BMR, and CC were positively correlated with FVC and FEV1 in men (*r* > 0, *p* < 0.05) (Table 4). Meanwhile, TBW, PC, MC, MM, FFM, SMM, BMR, CC, NC, and HC were positively correlated with FVC and FEV1 in women (*r *> 0, *p* < 0.05) (Table 5). The remaining body composition parameters did not correlate significantly with pulmonary function indicators.

**Table 4:** Age and smoking status-adjusted partial correlation analysis of body composition and pulmonary function in men. (1) *P ≤ 0.05, **P ≤ 0.01, ***P ≤ 0.001.

n	FVC		FEV1		FEV1/FVC	
	*r*	*p*	*r*	*p*	*r*	*p*
TBW	0.302	0.001***	0.297	0.001***	− 0.052	0.356
PC	0.301	0.001***	0.298	0.001***	− 0.051	0.366
MC	0.322	0.001***	0.324	0.001***	− 0.039	0.498
FM	0.003	0.952	0.007	0.897	− 0.030	0.598
MM	0.302	0.001***	0.297	0.001***	− 0.052	0.357
FFM	0.305	0.001***	0.300	0.001***	− 0.051	0.370
SMM	0.299	0.001***	0.295	0.001***	− 0.051	0.371
BMI	0.009	0.873	0.017	0.758	− 0.027	0.631
BFP	− 0.103	0.069	− 0.081	0.152	− 0.026	0.650
WHR	0.054	0.339	0.032	0.578	− 0.057	0.313
VFL	− 0.004	0.948	0.012	0.833	− 0.037	0.515
BMR	0.303	0.001***	0.300	0.001***	− 0.051	0.369
CC	0.136	0.016*	0.127	0.025*	− 0.048	0.394
WC	0.076	0.182	0.068	0.231	− 0.043	0.453
NC	0.098	0.084	0.076	0.180	− 0.048	0.398
HC	0.096	0.091	0.108	0.058	− 0.023	0.692

**Table 5:** Age and smoking status-adjusted partial correlation analysis of body composition and pulmonary function in women. (1)*P ≤ 0.05, **P ≤ 0.01, ***P ≤ 0.001.

*n*	FVC		FEV1		FEV1/FVC	
	*r*	*p*	*r*	*p*	*r*	*p*
TBW	0.233	0.001***	0.200	0.001***	− 0.063	0.180
PC	0.234	0.001***	0.202	0.001***	− 0.065	0.162
MC	0.235	0.001***	0.209	0.001***	− 0.049	0.299
FM	0.057	0.223	0.070	0.132	0.010	0.834
MM	0.233	0.001***	0.201	0.001***	− 0.064	0.171
FFM	0.234	0.001***	0.202	0.001***	− 0.063	0.174
SMM	0.233	0.001***	0.203	0.001***	− 0.061	0.195
BMI	0.030	0.523	0.053	0.254	0.011	0.810
BFP	− 0.055	0.237	− 0.002	0.961	0.043	0.363
WHR	0.009	0.847	0.020	0.667	0.041	0.378
VFL	0.005	0.914	0.044	0.348	0.028	0.552
BMR	0.234	0.001***	0.202	0.001***	− 0.064	0.173
CC	0.093	0.047*	0.104	0.025*	− 0.005	0.921
WC	0.068	0.147	0.078	0.094	0.015	0.744
NC	0.097	0.038*	0.094	0.044*	− 0.022	0.638
HC	0.117	0.012*	0.127	0.006**	− 0.018	0.702

**Figure 1:**
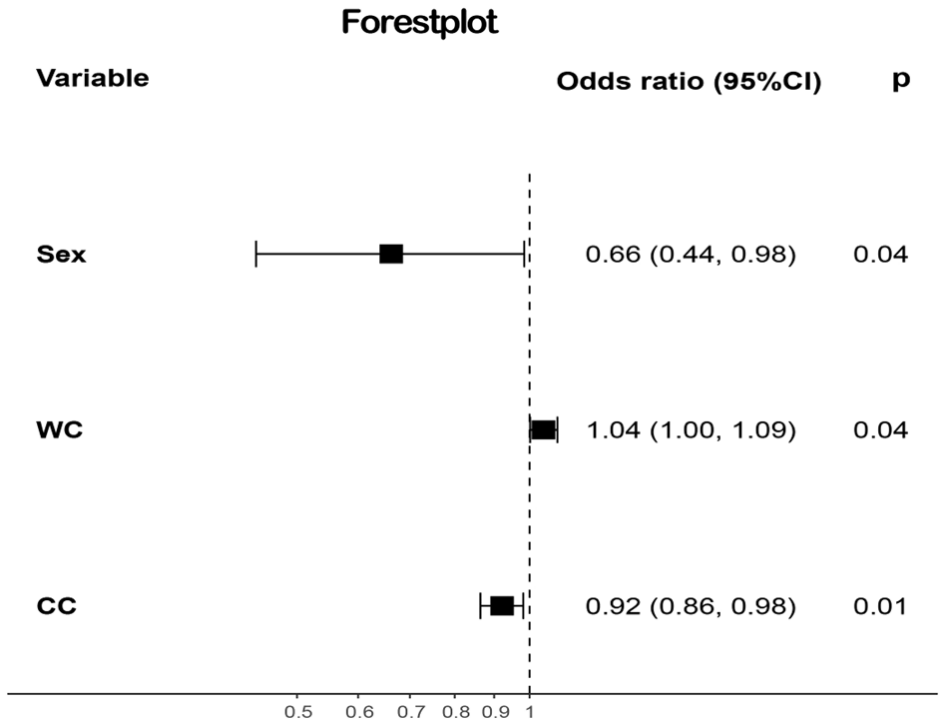
Logistic regression forestplot. (1) Sex, Weight, Height, WC, HC, NC, and CC were included in the stepwise regression analysis.

A multifactor logistic regression analysis was performed to determine whether lung function had decreased as the dependent variable and sex, weight, height, WC, HC, NC, and CC, which remained after we removed or combined independent variables with multicollinearity, as independent variables, using a stepwise regression method and drawing forest plots (Fig. 1). The results revealed that lung function declines more slowly in women than in men (odds ratio [OR] = 0.66, 95% confidence interval [CI] = 0.44–0.98, *p* = 0.04); increased CC (OR = 0.92, 95% CI = 0.86–0.98, *p* = 0.01) is a protective factor for reduced lung function, and conversely, increased WC (OR = 1.04, 95% CI = 1.00–1.09, *p* = 0.04) is a risk factor for reduced lung function. The results of the sensitivity analysis indicated that CC always had an impact on lung function, whereas sex and WC had an impact on different groups of lung function indicators (Fig. S1).

## Discussion

After controlling for the effects of age and smoking, TBW, PC, MC, MM, FFM, SMM, BMR, and CC positively correlated with FVC and FEV1 in men. TBW, PC, MC, MM, FFM, SMM, BMR, CC, NC, and HC positively correlated with FVC and FEV1 in women. In addition, the decline in lung function is slower in women than in men. CC increases as a protective factor against lung function. Increased WC is a risk factor for decreased lung function.

To the best of our knowledge, this is the first study to analyze the relationship between various body components and the main indicators of lung function. Similar to previously reported results, we identified that some body composition indicators were positively associated with lung function; for example, a 7-year longitudinal study has reported that a reduced FFM was associated with decreased lung function^20^. A study on athletes showed that FFM and muscle mass (MM) were positively and independently associated with FEV1 and FVC^21^. Health screening results for Korean residents showed that low MM was associated with low lung function, and SMI reduction was also independently associated with it^22,23^. In addition, we found that TBW, PC, MC, BMR, and CC were positively correlated with lung function in both men and women, whereas NC and HC were positively correlated with lung function in women and were not affected by age or smoking status. Understanding these connections can help individuals become more conscious of healthy lifestyles that balance body composition, thereby reducing the risk of declining lung function.

In a stepwise regression, we found that men were at a greater risk of reduced lung function, possibly because a greater proportion of men were former and current smokers, and smoking increased the negative impact on lung function^24^. Increased CC was a positive predictor of lung function, and the results were robust in sensitivity analyses. Several studies have concluded that CC is a good body shape indicator and positively correlated with pulmonary function in adolescents and adults^25,26^. WC is an important indicator for assessing the degree of abdominal obesity. The accumulation of abdominal fat restricts the respiratory movements of the lungs and interferes with respiratory function; therefore, increased WC is a risk factor for reduced lung function^27,28^.

Our study failed to identify correlations among other obesity-related indicators, such as FM, BMI, BFP, WHR, VFL, and lung function. This suggests that central obesity, rather than general obesity, is independently associated with reduced lung function and restricted pulmonary ventilation^29^, and obesity beyond a certain limit of the normal weight range may not significantly affect pulmonary function, as further judgment is required depending on the degree of obesity. Moreover, the TBW, PC, and MC in FFM were positively correlated with lung function indicators (FVC and FEV1) in men and women.

Nevertheless, this study had several strengths. First, we went beyond the traditional two-component model and included more precise and comprehensive body composition indicators to examine the relationship between body composition and lung function, rather than just obesity-related indicators, which was rare in previous studies. Second, we included a wide age range of male and female groups, analyzed men and women separately to obtain the relationship between their respective body composition and lung function, and controlled for confounding factors of age and smoking status. Increasing age not only increases body fat content and decreases skeletal muscle mass, whole-body water content, and mineral density but also has a functional effect on lung ventilation^30,31^. Therefore, our findings have more generalized credibility and reliability. Finally, we performed a bioelectrical impedance analysis to measure body composition indicators directly without the influence of reporting bias.

This study had some limitations. First, the study sample size was relatively small and limited to the Ningxia region; therefore, the applicability of its findings is somewhat limited, and a collaborative survey with a large sample from multiple regions should be conducted. Second, the number of participants with a decline in pulmonary function index (FEV1/FVC ratio) was small. This may have been affected by selection bias, as it was based on cross-sectional data obtained from a cohort study follow-up. Respondents should be carefully and rationally selected, and the study participants should demonstrate better cooperation to reduce the rate of invalid responses and lost interviews. Finally, because this was a cross-sectional study, we were unable to demonstrate a causal relationship between body composition and lung function. Cohorts will need to be established for further study.

In conclusion, the body components TBW, PC, MC, MM, FFM, SMM, BMR, and CC positively correlated with pulmonary function (FVC and FEV1) in both sexes. NC and HC were positively correlated with pulmonary function (FVC, FEV1) in women. Men had a higher risk of reduced lung function than that for women. Increased CC is a protective factor against decreased lung function, whereas increased WC is a risk factor for reduced lung function. The possibilities demonstrated by these results are important for assessing the effects of body composition on lung function.

## Methods

### Study population

We used cross-sectional survey data from a prospective cohort. In 2008 and 2012, two towns were randomly selected from the rural areas of Qingtongxia and Pingluo Counties in Ningxia, China. Two villages were randomly selected from each town, resulting in four administrative villages as the survey units. Questionnaires, physical examinations, and biochemical blood tests were administered to adult participants aged ≥ 18 years, and data were collected from a total of 2209 participants (1265 in 2008 and 944 in 2012). All the participants provided written informed consent.

A face-to-face survey of all participants was conducted from 2019 to 2020, resulting in a follow-up of 1,655 participants, a follow-up rate of 74.92%, and an average follow-up time of 9.75 years. The exclusion criteria included missing questionnaires, physical examinations, or blood biochemistry data at the baseline survey and follow-up (*n* = 390); people who died during the follow-up period (*n* = 138); people with chronic obstructive pulmonary disease, emphysema, lung cancer, or other lung-related diseases (*n* = 344); and people not in the age range of 30–75 years (*n* = 7). In total, 776 participants were included in the analysis. This study was approved by the Life Sciences Ethics Review Committee of Ningxia Medical University (2018-012, 2020-689). Written informed consent was obtained from all the participants. Our research was conducted in accordance to the Declaration of Helsinki.

### Measurements

#### Body-composition measurements

Healthcare professionals in this study measured body composition indicators using a body composition analyzer (InBody 370, Seoul, South Korea) that employs DSM-BIA.

The participants were required to fast, avoid alcohol consumption, and avoid exercise 8–12 h prior to the test. During the test, participants should have empty bowels and bladders, wear light clothing, remove metal ornaments, wipe the palms of their hands and feet with electrolytic wipes supplied with the instrument, stand barefoot on the electrodes of the footplate, hold the electrode part of the handheld handle with both hands and feet in close contact with the electrodes, relax the body, and drop the upper limbs naturally. After the tester entered the basic personal data, the computer measurement button was clicked to perform the measurement, and data related to the subject's body composition were recorded after the instrument readings stabilized.

In this study, the final analysis contained the following body composition indicators: TBW (L), PC (kg), MC (kg), FM (kg), FFM (kg), MM (kg), SMM (kg), BMI (kg/m^2^), BFP (%), WHR, VFL, BMR (kcal), CC (cm), WC (cm), NC (cm), and HC (cm).

#### Lung-function measurements

Pulmonary function parameters were measured by trained professional spirometrists, using a digital spirometer linked to a computer (ChestGraph HI-101; Tokyo, Japan). The instrument was calibrated before collection of data on lung function according to the instructions for use. After instructing the patient to sit still for 3 min, spirometry was performed with the patient wearing a nose clip, sitting up straight in a chair, wrapping the lips tightly around the port, slowly inhaling the maximum amount of air, and exhaling all air quickly and without stopping. From the flow–volume curve (F–V curve), it was evident that there was no hesitancy in the onset of expiration, expiratory flow spikes appear rapidly, and the extrapolated volume (Extrap v) was < 150 ml. No interruptions or leaks throughout the exertion breathing curve or the closed-flow inspiratory loop. The time-volume curves (T–V curves) demonstrated an expiratory plateau lasting up to ≥ 1 s. Measurements were performed at least three times, with an error of < 5% or < 200 ml between the two best measurements. Quality control to meet the standards of the operation specifications uses the best value as a value record. Among the lung function parameters measured, FVC, FEV1, and the ratio of forced expiratory volume in one second to forced vital capacity (FEV1/ FVC) were selected. The instrument automatically generates FEV1 prediction (FEV1pre) and FVC prediction (FVCpre) based on prediction equations adapted for Asian populations, and the prediction varies according to the characteristics of specific populations (age, height, sex, and ethnicity). Decreased lung function was defined as: FVC% < 80% was abnormal, FEV1% < 80% was abnormal, FEV1.0/FVC% < 70% was abnormal, and any one of the three tests below the standard value was defined as abnormal lung function^32^.

### Statistical analysis

In this study, continuous and normal variables are described as means (standard deviations), discrete and non-normal variables as median (25th percentile, 75th percentile), and categorical variables as frequency (percentage). Data demonstrated a normal distribution. The mean comparison between groups was performed by T-test or analysis of variance; data did not obey a normal distribution and were analyzed after log transformation or the Mann–Whitney U test was used to test for differences between groups, and rates were compared using the chi-square test.

After controlling for age and smoking status, a partial correlation analysis was performed to determine the relationship between body composition and lung function. A Pearson partial correlation analysis was used for continuous and normal data, and a Spearman partial correlation analysis was used for non-normal and rank data. Smoking status was classified as current smoker, former smoker, or nonsmoker, and stepwise regression analysis was performed to determine whether lung function had decreased as the dependent variable and body composition as the independent variable and a forest plot was drawn. The variance inflation factor (VIF) was used to test for multicollinearity, with a VIF of > 10 indicating strong multicollinearity. All statistical analyses were performed using the R4.2.1 software and RStudio. All tests were two-sided, and significance was set at *p* < 0.05.

### Supplementary Information

Supplementary Information.

## Data Availability

The datasets used and/or analyzed in the current study are available upon reasonable request from the corresponding authors (Faxuan Wang: faxuan203@163.com).

## Abbreviations

TBW: Total body water
PC: Protein content
MC: Mineral content
FM: Fat mass
FFM: Fat-free mass
MM: Muscle mass
SMM: Skeletal muscle mass
BMI: Body mass index
BFP: Body fat percentage
WHR: Waist to hip ratio
VFL: Visceral fat level
BMR: Basal metabolic volume
CC: Chest circumference
WC: Waist circumference
NC: Neck circumference
HC: Hip circumference
FVC: Forced vital capacity
FEV1: Forced expiratory volume in one second
FEV1/FVC: The ratio of forced expiratory volume in one second to forced vital capacity
