# Supplementary material for: Relationship between body composition and pulmonary function in the general population—a cross-sectional study in Ningxia
Source: Sci Rep. 2023 Oct 19;13:17877. doi: 10.1038/s41598-023-44486-9 (PMC10587154; doi:10.1038/s41598-023-44486-9)
Supplement: Supplementary file 1 — Supplementary Information. [file 41598_2023_44486_MOESM1_ESM.pdf]

Relationship between body composition and pulmonary function in the general population—A cross-sectional study in Ningxia

Yang-yang Pi[1,2], Wen-xuan Hu[1,2], Zi-ming Jiao[1,2], Peng-yi Hou[1,2], Yu-hong Zhang[1,2], Yi Zhao[1,2], Xiao-xia Li[1,2], Jing Yu[1,2] , Fang Chen[1,2], Jin-yun Jing[3] & Fa-xuan Wang[1,2]*

[1]School of Public Health, Ningxia Medical University, Yinchuan, 750004, People's Republic of China;

[2]Laboratory of Environmental Factors and Chronic Disease Control, Ningxia Medical University, Yinchuan, 750004, People's Republic of China;

[3]Ningxia Hui Autonomous Region Maternal and Child Health Care Hospital, Yinchuan, 750004, People's Republic of China.

*faxuan203@163.com

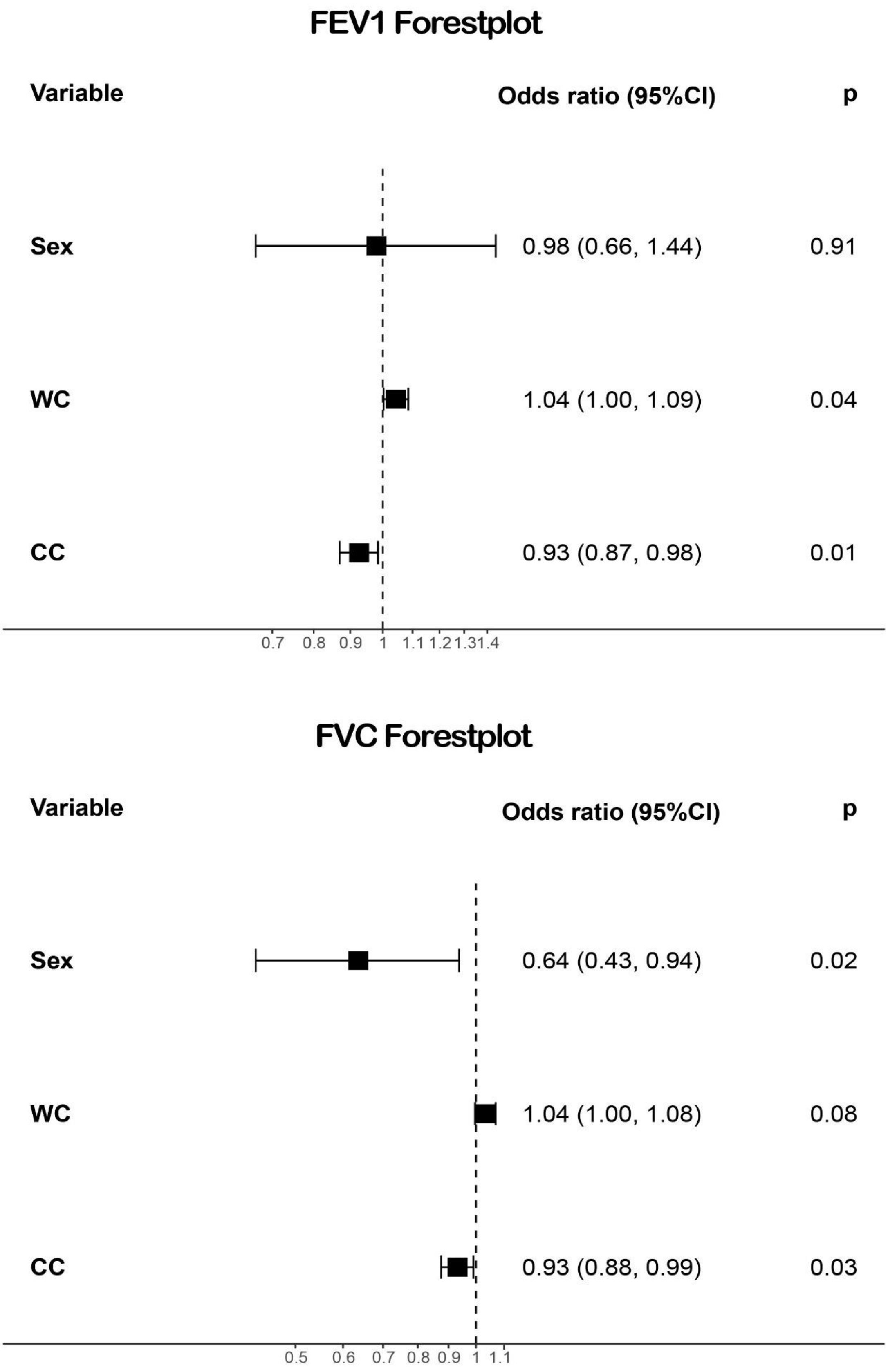

Figure.S1 Sensitivity analysis
